# Transformation of the *Cyanidioschyzon merolae* chloroplast genome: prospects for understanding chloroplast function in extreme environments

**DOI:** 10.1007/s11103-016-0554-8

**Published:** 2016-10-28

**Authors:** Maksymilian Zienkiewicz, Tomasz Krupnik, Anna Drożak, Anna Golke, Elżbieta Romanowska

**Affiliations:** Department of Molecular Plant Physiology, Faculty of Biology, University of Warsaw, ul. Miecznikowa 1, 02-096 Warsaw, Poland

**Keywords:** Stable chloroplast transformation, *Cyanidioschyzon merolae*, Chloramphenicol acetyltransferase, PEG, Biolistic bombardment

## Abstract

**Key message:**

We have successfully transformed an exthemophilic red alga with the chloramphenicol acetyltransferase gene, rendering this organism insensitive to its toxicity. Our work paves the way to further work with this new modelorganism.

**Abstract:**

Here we report the first successful attempt to achieve a stable, under selectable pressure, chloroplast transformation in *Cyanidioschizon merolae*—an extremophilic red alga of increasing importance as a new model organism. The following protocol takes advantage of a double homologous recombination phenomenon in the chloroplast, allowing to introduce an exogenous, selectable gene. For that purpose, we decided to use chloramphenicol acetyltransferase (CAT), as chloroplasts are particularly vulnerable to chloramphenicol lethal effects (Zienkiewicz et al. in Protoplasma, 2015, doi:10.1007/s00709-015-0936-9). We adjusted two methods of DNA delivery: the PEG-mediated delivery and the biolistic bombardment based delivery, either of these methods work sufficiently with noticeable preference to the former. Application of a codon-optimized sequence of the *cat* gene and a single colony selection yielded *C. merolae* strains, capable of resisting up to 400 µg/mL of chloramphenicol. Our method opens new possibilities in production of site-directed mutants, recombinant proteins and exogenous protein overexpression in *C. merolae*—a new model organism.

**Electronic supplementary material:**

The online version of this article (doi:10.1007/s11103-016-0554-8) contains supplementary material, which is available to authorized users.

## Introduction

*Cyanidioschyzon merolae* is an extremophilic red microalga that dwells in moderately high temperatures (40–56 °C) and highly acidic (between pH 0.2–4) environments (Ciniglia et al. 2004). Perhaps the most characteristic feature of this organism is its relatively simple anatomy. The *C. merolae’s* cell consists only of a nucleus, one mitochondrion and one chloroplast. Genomes of all three of these organelles were fully sequenced (Matsuzaki et al. 2004; Ohta et al. 1998, 2003). This simplicity can be utilized for genetic engineering and production of transgenic organisms, especially *via* site-directed mutagenesis, particularly interesting in physiological and structural studies of whole cells as well as intracellular organelles i.e. chloroplasts. Despite the apparent simplicity, only few protocols for stable nuclear transformation have been published up to date (Minoda et al. 2004; Imamura et al. 2009, 2010; Fujiwara et al. 2013, 2015; Miyagishima et al. 2014; Sumiya et al. 2015; Watanabe et al. 2014) and no successful, stable transformation of chloroplast have yet been reported. All of the nuclear transformation protocols relayed on introducing the wild-type *URA*5.3 (CMK046C) gene into *C. merolae* a spontaneous mutant (M4 strain), deficient in the UMP synthase gene (due to a frame-shift mutation) thus exhibiting uracil-dependent growth. The combination of the wild-type URA5.3 with uracil-deficient mutant sets up a limitation on *C. merolae* engineering to nucleus only. There have also been other, successful attempts to transform the chloroplast of a similar red alga (*Porphyridium* sp.) by application of an herbicide resistance gene (Lapidot et al. 2002). Recently, we have shown that a codon-optimized sequence of the *cat* gene, coding for chloramphenicol acetyltransferase can be used as a selectable marker for stable nuclear but also, potentially chloroplast transformation of *C. merolae* (Zienkiewicz et al. 2015). Here, we set out to establish the first protocol for stable chloroplast transformation of *C. merolae*, allowing us to perform a stable, site-directed mutagenesis of the plastid genome in the future.

In the following studies we have utilized the efficient expression of chloroplast encoded proteins to achieve high enough level of chloramphenicol acetyltransferase protein production, capable of providing sufficient protection of chloroplast ribosome machinery from chloramphenicol (Zienkiewicz et al. 2015), even in the apparently unfavorable environment of chloroplasts. The problem of the exogenous proteins instability, expressed in chloroplast has been encountered before (Birch-Machin et al. 2004; Sakamoto 2006), however little explanation has been given. On another hand, it was determined that specific N-terminal sequences may increase the half-life of chloroplast expressed proteins (Apel et al. 2010), however they were not incorporated into the *cat* sequence used in this study. The three-dimensional structure of proteins was also implicated in protein stability (Adam 2007), but next to nothing is known about the nature of these structural determinants of protein stability (Bock 2014).

The integration of a transgene with the plastid genome proceeds *via* homologous recombination and in general, plastid genes are not silenced by RNA interference. Additionally, *C. merolae* doesn’t possess the Dicer enzyme (Casas-Mollano et al. 2008), therefore the RNAi phenomenon doesn’t occur (Day and Goldschmidt-Clermont 2011). It has been presented in our previous work, that a stable nuclear transformant line with the *cat* gene fused with a signal peptide, directing the chloramphenicol acetyltransferase to the chloroplast, had allowed this organism’s cells to grow under high selective pressure of the antibiotic (Zienkiewicz et al. 2015). Therefore, we decided that the marker gene of our choice will be chloramphenicol acetyltransferase (CAT). The application of *cat* is highly advantageous over other antibiotic resistance genes, because *C. merolae* practically doesn’t generate spontaneous resistance to chloramphenicol. In case of other antibiotics, commonly used as chloroplast transformation markers, the rate of spontaneous resistance can be very high and even similar to the rate of chloroplast transformation (Day and Goldschmidt-Clermont 2011). As argued before (Zienkiewicz et al. 2015), the *cat* gene requires extensive nucleotide optimization. The codon usage analysis showed significant differences between nuclear and plastid codon frequencies, prompting a specific sequence optimization, to the most frequently occurring codons in the *C. merolae* plastid. The increasing number of scientific reports on various aspects of *C merolae* protein function and structure (Krupnik et al. 2013; Nilsson et al. 2014) has elevated this alga to a new model organism in photosynthetic research, hence the urgent need for production of plastid site-directed, homogenous, single-colony mutants.

## Results

**Fig. 1:**
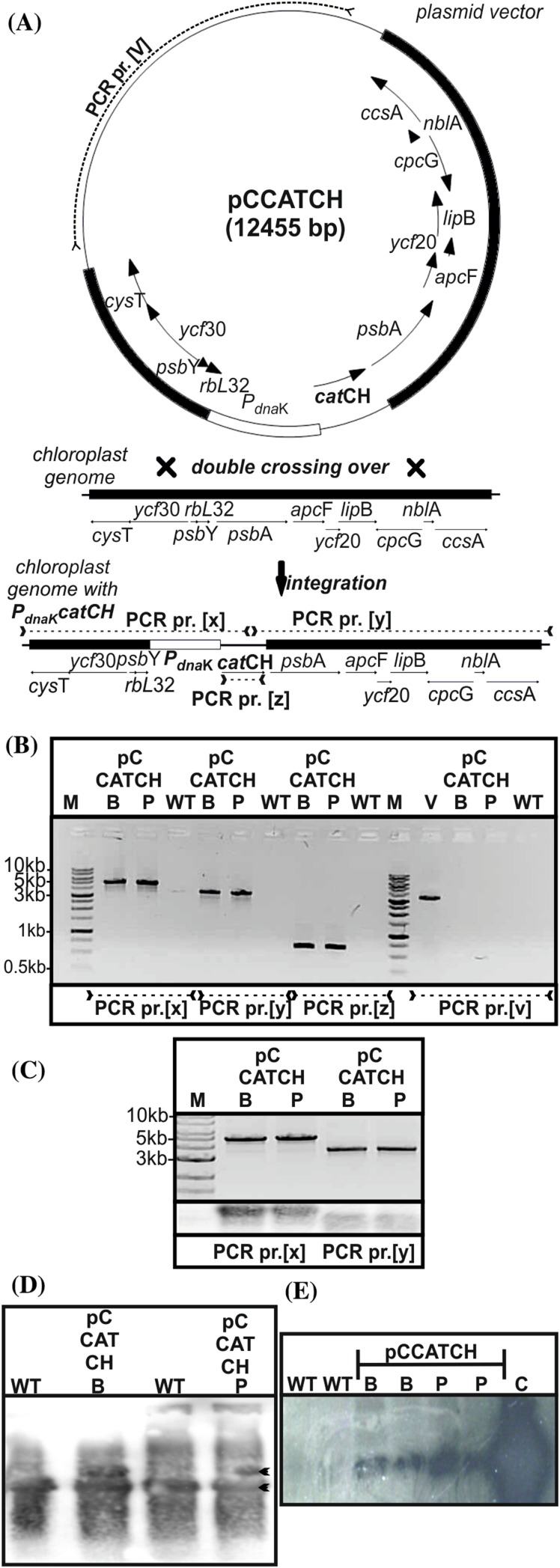
A schematic representation of a double homologues recombination event between transformation vector (pCCATCH) and chloroplast genome (**a**). The sequence of the *cat*CH gene, under control of the *C. merolae* chloroplast promoter P_*dna*K,_ (marked as *white, curved rectangle*) of the *dna*K gene, was integrated into the chloroplast molecule at the selected position between the *rpl*32 -*psb*A genes. The *solid black rectangles* represent the algal chloroplast sequence, also present in the transformation vector, flanking the *cat*CH cassettes and functioning as facilitators of double homologues recombination. **b** Verification of the insertion of the *cat*CH cassette into the *rpl*32-*psb*A region of the chloroplast molecule *via* PCR and subsequent agarose gel electrophoresis of the PCR products. The PCR reaction was performed on the total DNA, isolated from the wild type (*WT*) and stable chloroplast transformant lines (transformed by *B* biolistic bombardment or *P* PEG method). Two sets of primers were used: 5UTF-5UTR amplifying a 4612 bp long product (marked as x) and 3UTF-3UTR amplifying a 3655 bp long product (marked as y). These two PCR products encompass the regions between *cat*CH gene and fragments from beyond the upstream or downstream homologous region in the transformation vector. The presence of the *cat*CH was confirmed by a PCR reaction with catCHStuI-catCHKpnI primers pair amplifying a 672 bp long product (marked as z). To exclude the possibility of unwarranted, random integration of the vector or its continuous presence in the cell, a fragment of the plasmid *ori* region was amplified with the pABB20R-pABB20L primer pair, yielding a 3420 bp long product, only in case of the vector control, thereby confirming the absence of any residual plasmid (marked as v). *M* molecular marker. **c** Southern blot hybridization of PCR products generated by the 5UTF-5UTR and 3UTF-3UTR primer pairs with the CATCH probe (being the PCR product generated with catCHStuI-catCHKpnI primers pair). The total DNA, isolated from the stable chloroplast transformants with the integrated pCCATCH, obtained *via* biolistic bombardment (*B*) or PEG method (*P*) was used as a templates for PCR. The correct integration of the transformation vector was confirmed by the size of apparent bands (4612 bp and 3655 bp, see the *upper part*) and additionally by Southern blot hybridization with *cat* probe (the *lower part*). **d** The Southern blot hybridization of BglII-digested total DNA with the *psb*A probe. The total DNA was isolated from both lineages (*P, B*) of the transformed cells as well as the wild type. After digesting with BglII restriction enzyme the total DNA was loaded onto the agarose gel in equal amounts of 8 µg of DNA. The DNA was transferred onto a nitrocellulose membrane and hybridized with *psbA* probe. The transformant-derived *psbA* containing band was calculated to have the length of 4.2 kbp. The WT control fragment was calculated to form a 2.5 kbp band. The gel was transferred onto a nitrocellulose membrane and hybridized with an earlier prepared *psb*A probe. The semiquantitative results showed two bands (pointed out with *arrows*) in the *P* and *B* lines, representing the wild type ptDNA band and 1.8 kbp larger (containing P_dnaK_ *cat*CH cassette) recombined ptDNA band. The presence of both types of plastids demonstrated heterogeneity of the obtained transformant lineages. **e** The Southern blot hybridization of EcoRI-digested total DNA with the *cat*CH probe. The total DNA was isolated from both lineages (*P, B*) of the transformed cells as well as the wild type. After digesting with EcoRI restriction enzyme the total DNA was loaded onto the agarose gel in equal amounts of 5 µg of DNA. The *cat* containing band was calculated to have the length of 10 kbp. Additionally, a positive control was added by digesting the transformation plasmid with EcoRI and AatII. This fragment was calculated to range ~10 kbp band. The gel was transferred onto a nitrocellulose membrane and hybridized with an earlier prepared ^32^P enriched probe. The developed film shows no signal in the *WT* wells and one dominant signal in the transformed cells lines, confirming that the transformation vector had successfully recombined in a unique and single locus of the plastid without any unwarranted, illegitimate recombination with the genome or the plastid. The positive control is overexposed as the molar contribution of the *cat* sequence in the transformation vector is great deal higher than in the plastid

**Fig. 2:**
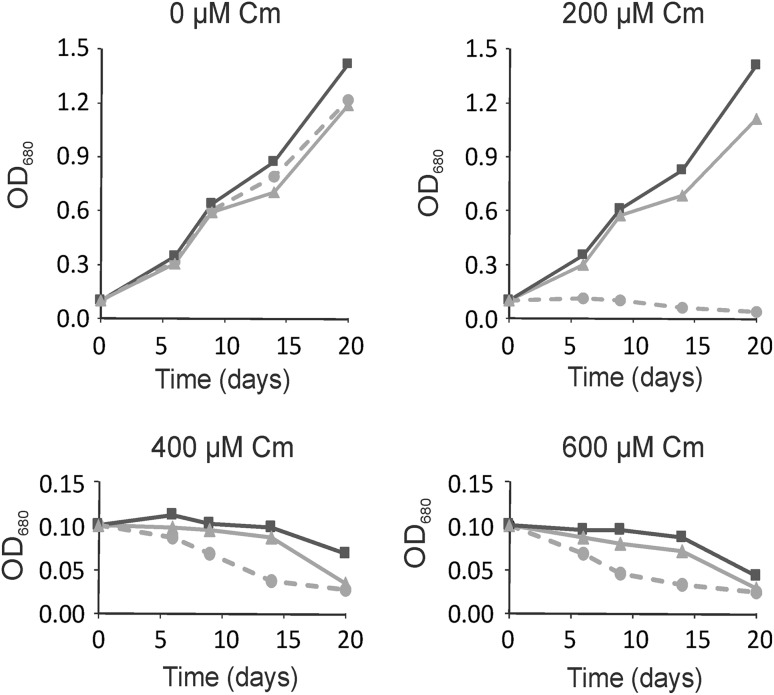
Growth dynamic of *cat*CH-integrated cells obtained by biolistic bombardment (B *dark, square* marker) and PEG-mediated method (P *gray, triangle* marker) vs. wild type (WT *light, dashed line*) *C. merolae* in rising concentration of chloramphenicol (0–600 μg/mL). Cultures were led for 20 days, under continuous light of 50 μmoles of photons m^−2^ s^−1^. OD measurements were taken at λ_680_ nm. Stable chloramphenicol resistant varieties could sustain growth in 200 μg/mL of chloramphenicol but not in any higher concentration (400 or 600 μg/mL) of chloramphenicol. Wild type cells growth was inhibited in all tested chloramphenicol concentrations

**Fig. 3:**
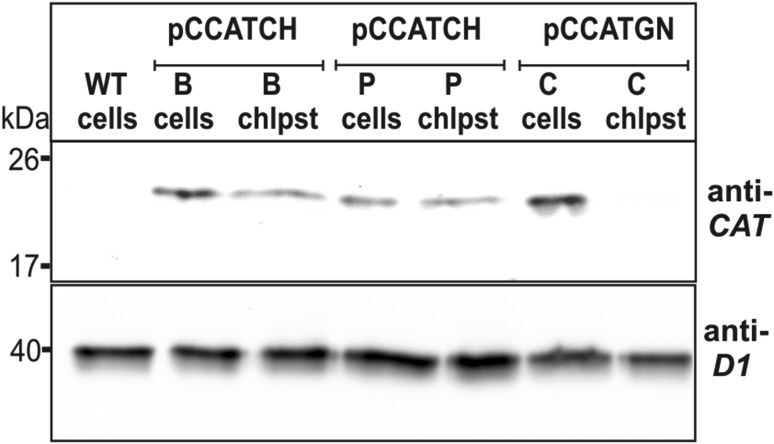
Western blot verification of the expression of chloramphenicol acetyltransferase in the chloroplast of stable chloroplast transformants lines. Transformed cells with pCCATCH by biolistic bombardment (*B*) and PEG method (*P*) were analyzed *via* WB. The presence of CATCH protein was tested in the whole cells (cells) and chloroplast fraction (chlpst) of both varieties. *C* The stable nuclear transformant pCCATGN with cytosolic localization of the CAT protein was used as a control. Samples were loaded on the SDS–PAGE gel in identical equivalents of chlorophyll *a*: 0.25 µg of Chl*a* for cells and chloroplasts. The abundance of CAT protein in chloroplasts was comparable with the cellular levels. Neither, the wild type (*WT*) cells nor the chloroplast fraction of the stable nuclear transformants contained detectable levels of the CAT protein. To confirm that equal amounts of material were loaded on the gel, a chloroplast protein D1 was detected with anti-D1 antibody (*lower part*)

**Fig. 4:**
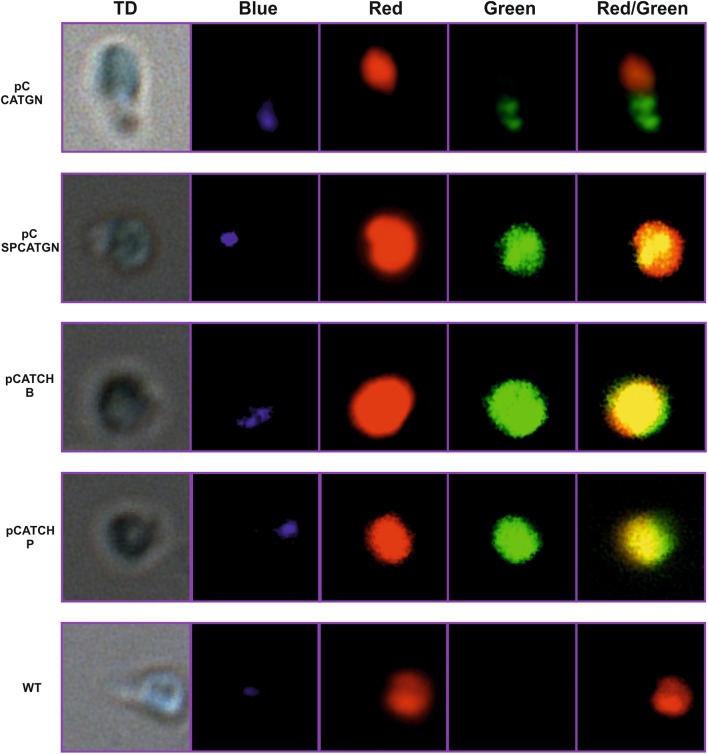
Immunocytochemistry analysis of the stable chloroplast *C. merolae* transformant cells, expressing CAT enzyme. Cells transformed with the pCCATCH (DNA delivered by *B* biolistic bombardment or *P* PEG method) were permeated with the anti-CAT antibody and labeled with green fluorescent dyed secondary antibody. Chromatin was visualized as blue fluorescence of Hoechst 33258 dye (Sigma-Aldrich, Germany). The natural emission of chlorophyll *a* was observed as red fluorescence. The stable nuclear transformant-cells with chloroplast location of CAT (transformed with pCSPCATGN) and cytoplasmic location (transformed with pCCATGN) were used as controls, while the wild type cells as the negative control. Cells were analyzed using a fluorescence confocal microscope (Nikon A1R MP). Strong green fluorescence of CAT protein was perfectly collocated with red fluorescence, stemming from the chlorophyll *a* in both (*B, P*) chloroplast-transformed cells as well as in control nuclear varieties. In case of the cytoplasm-located CAT nuclear variety, the green fluorescence was detected in cytoplasm. Additionally, blue fluorescence of chromatin never collocated with the res fluorescence of chloroplast, allowing to confirm a reasonably high resolution of presented pictures

The codon-optimization of the *cat* gene sequence was performed in accordance with the method described earlier (Zienkiewicz et al. 2015). The chloroplast-optimized *cat* gene sequence (GenBank KX056487), annotated *cat*CH, revealed 80% identity with the native *cat* gene (Tn9 from pACYC184 vector). Simultaneously it showed roughly 75% of identity with the previously optimized *cat*GN gene, used for the nuclear transformation (the alignment of both modified *cat* genes is attached in Figure S1 in Supplementary Material). The construction of the transformation vector was based on the pABB20 bacterial plasmid (Figure S3 in Supplementary Material). The *cat*CH gene sequence, together with the *dna*K (CMV163C) promoter sequence were integrated into a selected, c.a. 6 kbp long sequence of *C. merolae* chloroplast genome, encompassing *rpl*32 (CMV046C) and *psbA* (CMV047C) genes. The precise locus of the *cat*CH cassette (cassette comprises of the *cat* gene and P_*dnaK*_ promoter) integration was strategically chosen for the convenience of further mutagenesis of the *psbA* gene (Fig. 1). It was expected that long flanks at the 5′ and 3′ end of the *cat* cassette (here ~2 and ~4 kb, respectively) of identical sequence will facilitate a double homologues recombination event, resulting in the integration of the selectable marker gene into the chloroplast genome. The promoter of the *dnaK* gene (CMV163C) was chosen to ensure a stable and continuous expression of *cat*CH. This gene is expressed constitutively (Kanesaki et al. 2012), regardless of light exposure or cell cycle. *C. merolae* culturing was conducted in an identical fashion as described before (Zienkiewicz et al. 2015). The 5′UTR region preceding the *psbA* gene, containing the rbL32 stop codon, the *psbA* promoter as well as putative *rbL*32 terminator (Fig. 1) was reconstructed at the 5′ UTR of the *psbA* gene. For transformation of *C. merolae* cells, two separate methods were chosen: the PEG mediated DNA insertion (annotated as P) and the biolistic bombardment (annotated as B; details are described in the “Materials and methods” section). In both cases the transformed cells were suspended in MA2 medium without chloramphenicol pressure for 3 days, subsequently 200 µg/mL chloramphenicol was added and the culture was incubated in normal growth conditions for 3 months. The growth medium was exchanged every week. It was observed that after first 10–14 days the liquid cultures of the transformed *C. merolae* had changed their color from green into yellow, probably as a result of dying off of the non-transformed, chloramphenicol-sensitive cells. Further on, the cultures had slowly begun to green again. Roughly 3 months old cultures were diluted to OD_680_ 0.1 (10^7^ cells/mL) and 100 µL was spread on Petri dishes with MA2 medium, solidified by 0.4% of gellan gum and supplemented with 200 µg/mL chloramphenicol. Cells were gently rubbed in, with a sterile paint brush until a uniform layer formed on the surface of individual plates. Plates were sealed with a triple layer of parafilm and incubated in standard growth conditions, right-side-up for 1 month, then plates were inverted up-side-down and incubated until single colonies begun to appear. Up to 10 colonies were transferred to 20 mL bottles with fresh MA2 medium, supplemented with 200 µg/mL of chloramphenicol. Medium was refreshed at 2-weeks intervals. Selected single-colony cultures were analyzed further. Three months old, continuously grown cultures were used to inoculate fresh cultures at OD_680_ 0.1 and the growth rates were measured for both types of transformants (B-bombardment and P-PEG mediated DNA delivery) together with the WT as the control in selected concentrations of chloramphenicol (Fig. 2). It was observed, that both transformants were able to grow under 200 µg/mL of antibiotic, but not under any higher pressure of chloramphenicol (400 or 600 µg/mL; Fig. 2). The introduction of the transgene into the plastid genome did not impede the growth ability in the control conditions for neither of the two transformant cases. They were able to grow at the rates identical to this of the wild type without the antibiotic (Fig. 2). All tested concentrations of chloramphenicol were lethal for the wild type. Western Blot analysis of the isolated, intact chloroplasts, demonstrated presence of the CATCH protein in chloroplasts of stable transformant lines, obtained with both types of methods (Fig. 3). As the purity control of the intact chloroplast isolation, a stable nuclear transformant (pCCATGN) was taken (marked as C in Fig. 3). This variety is characterized by cytoplasm-located expression of chloramphenicol acetyltransferase (Zienkiewicz et al. 2015). The analysis of CAT protein levels, present in the chloroplast and the whole cell, showed that the chloroplast transformant (pCCATCH) exhibits similar concentration of CAT in cells as in isolated chloroplasts. However, for the nuclear transformant (pCCATGN) CAT was present in cells but wasn’t detected in isolated chloroplasts at all, thereby indicating cytoplasm localization of the CAT protein. To confirm that equal amounts of material were loaded onto the gel an additional western blot analysis was run. The levels of a chloroplast-specific protein D1 (encoded by *psb*A gene) were probed with the anti-D1 antibody (Agrisera, Sweden), yielding identical levels of D1 protein (Fig. 3, lower panel). The CAT protein localization was additionally assessed by indirect immunofluorescence analysis of whole transformant cells, obtained with both methods: PEG and biolistic bombardment (Fig. 4). As additional controls, stable nuclear *C. merolae* transformants, with cytosolic (pCCATGN) and chloroplast (pCSPCATGN) CAT localization were shown. The latter possesses an N-terminally fused signal peptide of the *apc*C gene (CMO250C), directing the CAT protein to the chloroplast, as previously described (Zienkiewicz et al. 2015).

The PCR analysis of the CAT gene insertion into the chloroplast genome (Fig. 1) confirmed, that the CATCH cassettes had been integrated with the chloroplast genomes *via* double homologues recombination. Four pairs of primers were used to analyze the localization of *cat*CH cassettes in the chloroplast genome. The annealing sites were designed to encompass all possible integration sites and to ensure that the amplification can occur only in case of proper double integration event. Schematic representation of the PCR analysis was drawn in Fig. 1. The DNA band, amplified with by the 5UTF-5UTR and 3UTF-3UTR primer pairs, composed of a *cat* sequence fragment as well as the 5′ and 3′ chloroplast-genome sequences, absent in the transformation vector. The corresponding products (x and y respectively, Fig. 1) confirmed that a case of correct double homologues recombination did occur. The z product (Fig. 1) amplified the *cat*CH sequence and served as an internal control, aiming at ascertaining that the identical amount of PCR template was used for all reactions. In order to exclude the possibility of unwarranted and random integration of the transformation plasmid with the genome we have attempted to generate a product (v, Fig. 1) by amplifying the *ori* region of the vector. No V-product was generated, for either of strains, thereby ensuring that no event of “illegal” integration have occurred, nor any copy of the free transformation plasmid was harbored by *C. merolae* cells. A similar PCR analysis has been used before as a confirmation of the homologues recombination event between a CAT cassette and the chloroplast DNA molecules in *Phaeodactylum tricornutum* (Xie et al. 2014). The Southern hybridization (Fig. 1) of PCR generated x and y bands, using the CATCH probe, confirmed the presence of CAT sequence within the bands thus the specificity of the obtained bands. Subsequently, the proper integration of the CATCH cassette with the plastid molecule was confirmed by Southern blot hybridization of the total DNA and visualized with CAT-specific, radiolabeled probe (Fig. 1). The total DNA was digested with EcoRI restriction enzyme to form 10 kbp long DNA fragments encompassing the CATCH sequence and a fragment of the plastid. The transformation plasmid (as a positive control) was digested with EcoRI and AatII to form 10 kbp long fragment. We observed a single band in both transformed lines and with no detectable signal in the WT line. A strong signal in positive control was recorded. The results indicate that the selected transformant lineages did undergo a specific homologues recombination event, without unwarranted, random integration with the plastid nor the genome. Since plant chloroplasts possess 10–100 copies of the plastid molecule, it was necessary to estimate the efficiency plastid replacement and level of heterogeneity of chloroplast haplotypes, subjected to a long-term (3 months) selectable growth. For that purpose, the total DNA was isolated from both: B and P lineages as well as from the WT. Further, the DNA was digested with BglII restriction enzyme (Thermo, USA), cutting the plastid in the proximity of 3′ and 5′ ends of the *psbA* gene. Separated by electrophoresis DNA fragments were blotted onto a membrane and probe with *psbA*-probe (Fig. 1). The results of semiquantitative Southern blot confirmed that both haplotypes of the plastid are present within the transformed cells: the wild type as well as the recombined plastid, roughly 1.8 kpb larger. Finally, the total RNA was isolated from both: B and P lineages as well as from the WT and probed for *psbA* and *cat* genes. It was observed that the WT and both transformants exhibited identical size of *psbA* mRNA, however *cat* mRNA was present significantly below the *psbA* size, only in the transformed lineages (Figure S4, Supplementary Material), suggesting that *psbA* and *cat* were transcribed on speared strands of mRNA.

## Discussion

The ability to express exogenous proteins from the plastid genome has attracted a considerable attention (Maliga 2004; Daniell et al. 2009; Bock 2007) due to its capacity to accumulate large quantities of foreign proteins. This propensity is particularly advantageous for biotechnological applications (Meyers et al. 2010), as plastid protein expression may reach up to 70% of leaf or cell protein content (Daniell et al. 2009). *C. merolae* is known to grow in high ambient temperature of 42 °C and higher but it tolerates, reasonably well, temperature as low as 37 °C, therefore it might be well suited for human proteins production, requiring the precise human body temperature for maturation. In addition, this alga possesses a set of N-glycosylation enzymes that can be engineered to “humanized” potential biopharmaceutics and no enzymes leading to formation of deleterious N-glucans (Mathieu-Rivet et al. 2014). This could potentially decrease the immunogenicity of such obtained recombinant proteins or facilitate the formation of human-compatible and highly active N-glycosylated proteins, for its use as therapeutics. Other potential application is production of strains expressing high-yield of endogenous recombinant proteins for physiological and structural research in the field of light energy conversion, photosynthesis and chloroplast function. Similarly, production of site-directed mutants may pose an important tool for disrupting or otherwise altering genes of interest in particular metabolic pathways, enabling research aimed at probing into these problems. The introduction of heterogeneous *cat* gene as the selectable marker has several important advantages over phototrophy or metabolism restoration (León et al. 2007). Most importantly, the CAT expressing cassette can be introduced into any locus on the plastid and selection of the transformed lineages can be carried out on chloramphenicol-enriched and solidified MA2 medium on Petri dishes, additionally *C. merolae* practically does not generate spontaneous resistance to chloramphenicol. As argued before (Zienkiewicz et al. 2015; Li et al. 2011), chloramphenicol inhibits protein translation in bacterial and chloroplast ribosomes. Eukaryotic and mitochondrial ribosomes are largely or entirely imperious to chloramphenicol toxic effects. Even though, the CAT resistance protein seems to be very useful selectable marker, only few reports of successful chloroplast transformations using CAT as selectable marker were reported (Li et al. 2011; Tang et al. 1995). Chloramphenicol acetyltransferase (EC 2.3.1.28) is a bacterial enzyme, functioning in approximately neutral pH of bacterial cytoplasm (pH 7.6–8, with 50% activity at pH <5 and >9; Thibault et al. 1980) and 35 °C optimum temperature (with 20% activity at 30 °C and 55% at 45 °C; El-Gamal et al. 2001). The acidity of chloroplast stroma is known to sustain approximately stable pH of 7.4–7.8 in dependence on irradiation or cell cycle (Lee and Kugrens 2000), constituting seemingly perfect conditions for CAT to function. Indeed, our observations suggest that the expressed CAT enzyme exhibits very high activity, providing effective protection to the chloroplast’s ribosomes from chloramphenicol, even at concentration as high as 400 µg/mL. Perhaps the molecular structure or enzyme kinetics could be particularly susceptible to the presence of ROS, temperature or pH variation, what would diminish the activity or yield of the overexpressed enzymes. Unfortunately, the influence of the chloroplast environment on the recombinant proteins activity and stability remains largely unknown, setting up further limits to the scope of expressible proteins in this organelle.

In this report we described an innovative procedure for stable chloroplast transformation of *C. merolae*, utilizing the chloramphenicol acetyltransferase (*cat*) gene, codon-optimized for the chloroplast translation machinery of *C. merolae* as the selectable marker, under the *dnaK* promoter region. We showed, that PEG-mediated and biolistic bombardment based DNA-delivery methods, can be routinely used for plastid transformation of this alga. Unfortunately, our procedure did not allow us to provide any meaningful statistics, regarding the efficiency of transformation. The transformed algae cultures were incubated in a liquid medium, allowing for a selective growth of the resistant lineages, thereby inadvertently losing the information on transformation efficiency. However, up to date we have performed 11 PEG-mediated chloroplast transformations and ten of them were successful. The biolistic bombardment technique seemed to be more unpredictable and yielded only four positive transformations, in over 20 undertaken attempts. The presence of chloramphenicol acetyltransferase enzyme in the chloroplast was confirmed *via* western blotting (Fig. 3) and indirect immunofluorescence (Fig. 4). The PCR analysis combined with the Southern blot hybridization confirmed that homologues recombination between the plasmid vector and the chloroplast genome did occur (Fig. 1). Simultaneously, we have excluded any random integration with the plastid or the genome by Southern blot hybridization of the total DNA with a radiolabeled probe (Fig. 1). It is commonly accepted, that the illegitimate recombination results in a random integration of the whole molecules, in example: as the case of transposomes integration or the lambda phage integration (for review please see Würtele et al. 2003). The lack of the any vector remnants stands in an agreement with the earlier observation, stating that the site-directed homologues recombination occurs, predominantly, in plastids (Daniell et al. 2002; Verma and Daniell 2007). The plasmid construction required that the P_*dna*K_ *cat*CH cassette would have been terminated with the complete sequence, separating *rbL32* and *psbA* genes, containing putative terminator of the *rbL32*. A failure to terminate the transcription of *cat* resistance gene could adversely influence transcription of the following *psbA* gene and modify the chloroplast or photosynthetic activity in unpredictable way. To peer into this problem the total RNA was isolated form WT and both types of transformants and next probed for presence of *psbA* and *cat*CH mRNA (Figure S4). It was observed that the position of *psbA* and *cat* mRNA had differed significantly, allowing to conclude that the P_*dna*K_ *cat*CH cassette had fully functional terminator sequence and was located on a different mRNA strand than the *psbA*. This in turn implied that the *psbA* transcription most likely wouldn’t be hindered by transcription of the preceding *cat* gene. The growth dynamics, presented in Fig. 2 showed, that stable chloroplast-modified lines were capable of normal growth under selectable conditions with 200 µg/mL of chloramphenicol, a concentration lethal for wild type cells. This level of resistance to chloramphenicol was recorded for 3 months-old lineages. We anticipated, that the resistance level will increase over time, due to rising homogeneity of the modified plastid genome. The gradual increase of chloramphenicol pressure will, presumably, select for the highest expressers of CAT as the most prevalent haplogroup. Indeed, our latest results showed that a 6 months old culture was capable of resisting up to 400 µg/mL chloramphenicol (Figure S2, Supplementary Material). The multiplicity of plastid molecules in a chloroplast required that the efficiency of plastid replacement had been assessed. The total DNA was digested with BglII restriction enzyme to form 2.5 kpb long DNA fragments of wild type ptDNA as well as 4.2 kbp long recombinant ptDNA and probed for the presence of *psb*A gene (Fig. 1). The results of a semi-quantitative Southern blot exhibited both versions of plastid in the transformed lineages, pointing out heterogeneity of the obtained transformants. Heterogeneous haplotype of transformed chloroplasts had been reported before (Liu et al. 2007). There were also few cases, where the authors had postulated complete homoplasmy of the transformed chloroplasts (Li et al. 2011; Sidorov et al. 1999; Davarpanah et al. 2009), as well as cases where the heterogeneity of plastid population had not been assessed (Xie et al. 2014). We assumed that further growth, under stepwise increasing antibiotic pressure would have been able to select homoplastic lineages. Apparently, the attained CAT expression level was more than enough to provide protection for the ribosomal machinery of the chloroplast as well as a collateral protection to mitochondrial and cytoplasmic ribosomes and in effect contributed adversely to transformant selection process. Perhaps chloramphenicol was consumed too rapidly, allowing the low CAT expressers to survive for long enough so that, in the steadily decreasing selective pressure (the effect of CAT activity) they would have been able to resume their normal cell-proliferation cycle. Chloramphenicol (Sigma-Aldrich, Germany) is also sensitive to intense light irradiance as well as other factors like pH or temperature, these could contribute to unexpectedly fast decay of antibiotic concentration and in effect, dissipation of the selective pressure. Random distribution of heterogeneous pool of plastid molecules during chloroplast division would have favored the wild type restoration as the most prevalent haplotype in conditions of low or lack of chloramphenicol pressure. It is also possible that the integration of the transgene with a plastid molecule disabled the following gene (*psbA*) or caused other damage in the cell. In that case the presence of the WT plastid would be mandatory for cells to proliferate and state of transgenic homoplasmy could never be achieved as both types of plastid would coexist in a state of balanced heteroplasmy (Svab and Maliga 1993), allowing for sufficient cell proliferation and protection from chloramphenicol. Yet it is important to state that a long stretch of DNA, preceding the *psbA* sequence and containing putative promoter had been restored (and confirmed by DNA sequencing) what should, in principle, allow for normal function of that gene. Since the native sequence of *dnaK* gene promoter was used as the *cat* promoter, it might be that there was an unwarranted event of internal recombination within the transformed plastid (between the doubled *dnaK* promoter sequences), removing large fragments of ptDNA and effectively rendering it inviable. Further work is needed to ascertain the homoplasmic state of the transformed lineages, either by continuous selection in even higher concentration of chloramphenicol and more frequent (i.e. daily) medium exchange or by introduction of secondary marker i.e. GFP fused CAT enzyme, useful in cell sorting by FACS.

The overall resistance level of stable chloroplast transformants was significantly lower in comparison with the resistance level recorded in stable, nuclear, *cat*-harboring cells (up to 600 µg/mL). Simultaneously, we also proved that the chloramphenicol isn’t toxic for *C. merolae* mitochondrial machinery within the tested range (up to 400 µg/mL), most likely due to differences in the structure of ribosomes in these two organelles or collateral protection provided by chloroplast-located CAT enzyme (Zienkiewicz et al. 2015). The presented data showed, that an exogenous protein can be expressed in *C. merolae* chloroplast and the application of a constitutive promoter (P_*dna*K_) ascertained reasonably high and stable CAT-enzyme production rate. The combination of CAT as the selectable marker with chloramphenicol selectable pressure may provide a simple mechanism of introduction of point mutations, deletions or means to overproduce an entire exogenous protein in *C. merolae* chloroplasts. We have presented here the first and reliably working method for stable chloroplast transformation of *Cyanidioschizon merolae*. It delivers a powerful tool for genetic engineering within the chloroplast of this alga, helping to elucidate the structural and physiological aspects of this, ever more important model organism.

## Materials and methods

### Cell cultures

*Cyanidioschyzon merolae*, 10D (NIES-1332, Unialgal, Clonal and Non-axenic) strain was obtained from Microbial Culture Collection (mcc.nies.go.jp, Tsukuba, Japan) and was used throughout this study. Cells were grown in MA2 liquid medium (Minoda et al. 2004) in a glass vessel under continuous white light (50 μmol photon m^−2^ s^−1^) at 42 °C or on Petri dishes filled with MA2 medium, solidified by addition of 0.4% gellan gum (Phytagel™, Sigma, Germany) (Minoda et al. 2004) or 0.75% agar (Basica LE, Prona, EU).

*Escherichia coli*, strain DH5α (genotype: F−Φ80*lac*ZΔM15 Δ(*lac*ZYA-argF) U169 *rec*A1 *end*A1 *hsd*R17 (rK−, mK+) *pho*A *sup*E44 λ− thi-1 *gyr*A96 *rel*A1) were used for construction of transformation vectors (Hanahan 1983). Bacterial cells were cultured in liquid LB medium (1% Bacto tryptone, 0.5% yeast extract, 1% NaCl at 37 °C) or on Petri dishes with LB medium solidified by addition 1% agar. For selection of transformed cells, medium was supplemented with Kanamycin (30 µg/mL).

### Construction of plasmids used in this study

The pCCATCH plasmid (12,455 bp, a derivative of pABB20 vector; Bartosik et al. 2012): The transformation vector pCCATCH for stable transformation of chloroplast, containing 5′ UTR and 3′ UTR of *C. merolae psb*A (CMV047C), separated by *cat*CH cassette. The expression of the *cat*CH cassette was driven by *dna*K (CMV163C) promoter. Construction: The chloroplast genome sequence of *C. merolae* (coordinates 27,580–33,717 bp) was amplified by PCR with primers: NpsbAFor NpsbARev and Phusion II polymerase, then cloned into blunt ended vector with T4 Klenow NcoI site of pABB20. The plasmid was named pABB20Chl. To introduce the P_*dna*K_ *cat*CH cassette into pABB20Chl, a secondary plasmid pGFPP_*dna*K_ *cat*CH psbApr was constructed in following steps: the 1085 bp promoter region of the *dna*K gene (coordinates 106,558–107,643 bp) was amplified with primers dnaKSalI dnaKKpnI carrying SalI and KpnI restriction sites at the 5′ and 3′ ends respectively and cloned into pGFPuv vector (Clontech Laboratories, Inc., USA). The obtained plasmid was named pGFPuvP_*dn*aK_. Next, the optimized *cat*CH gene was amplified by primers: catCHKpnI and catCHStuI carrying KpnI and StuI restriction sites at 5′ and 3′ ends respectively and cloned throughout the KpnI and StuI restriction sites into the pGFPuvP_*dna*K_. The functional promoter of the *psb*A gene was restored after introduction of P_*dna*K_ *cat*CH cassette *via* the ScaI site in *psb*A promoter sequence. The restoration was achieved by PCR reaction with 48 bp synthetic fragments of the *C. merolae psb*A promoter region (coordinates 29,397–29,444 bp, labeled as recpsbAf and recpsbAr, complementary to each other). The constructed plasmid pGFPuvP_*dna*K_ *cat*CHP served as a matrix for PCR with primers dnaKSalI–psbArec which generated DNA fragment containing P_*dna*K_-*cat*CH-psbA-promoter-restored fragment, latter recloned into the pABB20Chl vector *via* ScaI restriction site, generating functional transformation vector pCCATCH with restored psbA promoter (see Figure S3 in Supplementary Material). Coordinates refer to chloroplast molecule Acc No. AB002583.1. The full sequence of the pCCATCH transformation vector was submitted to GenBank and is available under the Accession number KX056487.

Plasmids pCCATGN (13,770 bp) and pCSPCATGN (13,950 bp) were used as a control in indirect immunofluorescence and fluorescence microscopy, their construction was described before (Zienkiewicz et al. 2015).

### List of primers used in this study

The sequence of primers used in this study was presented in Supporting tables: Table S1.

### Enzymatic manipulations of DNA

All enzymatic manipulations on DNA, such as restriction digestion, blunting of cohesive termini using T4 Polymerase or the Klenow fragment or ligation were performed according to protocols supplied by the manufacturers.

### PCR

PCRs were performed according to manufactures protocols, supplied with the Phire Plant Direct PCR kit containing, Plant Phire Hot Start II DNA Polymerase (Thermo Fisher Scientific Inc., Waltham, USA) or DreamTaq DNA Polymerase, (Thermo Fisher Scientific Inc., USA).

### Plasmid DNA purification

Plasmid DNA purification was performed with the Extrectme Plasmid DNA Kit, Gdańsk, Poland.

### Transformation of *C. merolae*

Transformation of *C. merolae* was performed by either of the following procedures: PEG-mediated transfection or by biolistic bombardment with the PDS-1000/He Biolistic Particle Delivery System (Bio-Rad, USA). PEG-mediated transfections were performed as described before (Ohnuma et al. 2008) with the following modifications. A freshly started, overnight grown 100 mL of *C. merolae* culture at OD_750_ 0.4 was spun down at 2000×*g* for 5 min in 40 °C in a table-top centrifuge (5810 R, Eppendorf) and resuspended in 100 mL of MA-I buffer (20 mM (NH_4_)_2_SO_4_, 2 mM MgSO_4_, 1× trace elements at 40 °C). Then spun down again and resuspended in 500 µL of MA-I. The transformation mixture was prepared as follows: 20 µg of the plasmid was diluted in 400 µL of the MA-I buffer and mixed with 100 µL of cell suspension, subsequently 500 µL of 60% PEG (w/v) was added to final PEG concentration of 30% (w/v). The transformation mixture was incubated for 5 min at room temperature, then transferred to 50 mL of warm MA2 medium and incubated overnight in normal growth conditions. The following day the culture was spun down in 40 °C at 2000×*g* for 5 min and resuspended in 50 mL of fresh MA2 medium to wash off the remaining PEG. The culture was led for 3 days in normal growth conditions before the selectable conditions were introduced (see below).

The biolistic bombardments with the PDS-1000/He Biolistic Particle Delivery System were performed, in principle, according to the manufacturer manual. The 1550 PSI rapture discs and 0.6-micron gold microcarriers were chosen. Golden microcarriers, at stock concentration of 60 mg/mL were prepared as follows: 30 mg of microparticles was suspended in 1 mL of 70% ethanol (v/v) and vortex vigorously on a platform vortexer for 5 min, subsequently the suspension was incubated for 15 min at RT. Further, the suspension was spun down at 12,000×*g* in a microfuge for 5 s and ethanol was removed from above pelleted golden microparticles. This step was repeated three times to ensure proper washing off of any storage buffer. Next, the same washing procedure was applied further three times with 1 mL of sterile water. After the third wash, golden microcarriers were suspended in 500 μL of sterile 50% (v/v) glycerol. At this step the suspension can be stored in 4 °C until further use. The amount prepared, is sufficient for 60 bombardments, using 500 μg of the microcarriers per bombardment. Coating the microcarriers with DNA proceeds as follows: suspension of 3 mg of washed microparticles, sufficient for six repetitions of bombardment, was vigorously vortex for at least 5 min. Then, 5 µL of 1 µg/µL transformation plasmid was added, together with 100 µL of 2.5 mM CaCl_2_ and 2.9 µL of 1.38 M spermidine (Sigma-Aldrich, Germany) to continuously agitated microcarriers and vortexed for 2 min, followed by incubation on ice for 15 min. In the meantime, the mixture of microparticles with DNA was shortly vortexed in 3-min intervals. After incubation, the suspension of microparticles with DNA was shortly centrifuged (30 s at 12,000×*g*) and washed twice with 200 µL cooled (−20 °C) 100% ethanol. Finally, the microparticles already coated with plasmid DNA, were resuspended in 60 µL 100% ethanol. For every cycle of bombardment 10 µL of the suspension was spread on the rupture disc. The excess ethanol was evaporated by drying the macrocarriers in RT.

Biolistic bombardment was carried out as follows: a Petri dish with algal culture, grown to confluence (1–2 months) on MA2 medium solidified by addition of 0.75% agar, was placed in the distance of 9 cm from the rupture disc assembly. We used the 1550 psi rapture discs for shooting. Successively, the transformed algal cells were scraped off the plate surface, in a sterile fashion, with a spatula and resupended in 100 mL liquid MA2 medium. The culture was incubated in 42 °C, under normal growth conditions (at least 50 µE) for 3 days. In case of PEG-mediated transformation cells were transferred into 50 mL of MA2 and incubated in 42 °C under normal and identical growth conditions. On the fourth day chloramphenicol was added to the final concentration of 150 µg/mL. The selectable growth of transformed *C. merolae* culture was conducted for further 3 months. Every 7 days *C. merolae* cells were spun down (2000×*g* for 5 min at 40 °C) and resuspended in fresh MA2 medium supplemented with chloramphenicol (150 µg/mL). After 21 days the concentration of chloramphenicol was increased up to 200 µg/mL. Further, the *C. merolae* cells were plated with a sterile paint brush on Petri dishes with solid MA2 (0.4% gellan gum) supplemented with chloramphenicol at final concentration of 200 µg/mL and sealed with triple layer of parafilm. Plates were incubated for at least 4 weeks until single colonies appeared. Single-cell colonies (up to ten colonies) were transferred to 20 mL flasks of MA2 medium with 200 µg/mL of chloramphenicol. The culture was led for several months with medium exchange every 2 weeks. In parallel, the same cultures were led in step-wise (extra 50 µg/mL for every step) increasing concentration of chloramphenicol.

### *E. coli* competent cells

Preparation of *E. coli* competent cells and transformation was carried out as described earlier (Sambrook et al. 1989). Electrocompetent cells were prepared according to manufacturer instructions of GenePulser apparatus (BioRad, USA).

### Isolation of intact chloroplasts from *C. merolae*

Chloroplasts were isolated as described earlier (Minoda et al. 2010) with subsequent modifications (Zienkiewicz et al. 2015). The purity of intact chloroplast isolation was estimated by fluorescent microscopy, as described before (Zienkiewicz et al. 2015) and established to be over 97% pure.

### SDS–PAGE and immunoblotting analysis

Mutants and control cells were harvested by spinning down for 5 min at 2000 × g and resuspended in the resuspension buffer (20 mM Hepes-NaOH pH 7.6, 5 mM EDTA and 330 mM sucrose). Chlorophyll concentration was quantified spectrometrically (UV-1800 Shimadzu, Japan) by absorption measurement at λ_663_ of chlorophyll extracted with 80% (v/v) acetone. Numerical values were derived from Beer–Lambert equation, extinction coefficient ε = 86.3 (l g^−1^ cm^−1^) and the dilution factor (×200). Cells and chloroplast samples were solubilized in denaturing buffer (0.25 M Tris–HCl (pH 6.8), 0.4% (w/v) SDS, 10 M urea, 2% (v/v) 2-mercaptoethanol and 20% (v/v) glycerol) and mixed together in 1:1 (v/v) ratio. Proteins were separated on 15% gels by Laemmli-type SDS–PAGE method (Laemmli 1970). Gel wells were loaded with 0.2–1 μg of Chl*a* and run under constant voltage (75 V). Following electrophoresis polypeptides were electro-transferred on PVDF-membrane (Towbin et al. 1979) and probed with rabbit anti-CAT (specific to chloramphenicol acetyltransferase) antibodies (Sigma-Aldrich, Germany) or anti-D1 (Agrisera, Sweden). Bands specifically binding the probe were visualized by enhanced chemiluminescence method according to standard procedures using ChemiDoc System (Bio-Rad, USA).

### Nucleic acid isolation from *C. merolae* cells

DNA was isolated from *C. merolae* using standard CTAB in Situ Hybridization procedure described earlier (Schwarzacher and Heslop-Harrison 2000) and RNA isolation was performed by conventional methods (Fujiwara et al. 2009).

### Southern blot analysis

The transfer of the DNA from the agarose gel to a nitrocellulose membrane was performed by a conventional alkaline method using 20× SSC buffer (3 M NaCl, 0.3 M sodium citrate, pH 7.0). DIG-labeled DNA fragments were prepared by PCR using DIG Probe Synthesis Kit (Roche) with specific primers (catCHStuI and catCHKpnI hybridizing with the CATCH sequence or psbA_F and psbA_R primers hybridizing with the psbA sequence) and used as hybridization probes. The DIG was detected with alkaline phosphatase (AP)-conjugated anti-DIG antibody (Roche) and CDP-Star (Roche). The signals were visualized with the luminescent image analyzer ChemiDoc XRS+ System (Bio-Rad, USA) or X-ray film (Kodak BioMax XAR Film, Sigma-Aldrich, Germany) was exposed to the membranes for 24–48 h and developed with the Cerastream Kodak developer solution (Sigma-Aldrich, Germany).

### Radioactive Southern blot analysis

Equal amounts of total DNA (5 µg), isolated form transformed lineages, were digested with EcoRI and the positive control (the transformation plasmid) was digested with EcoRI and AatII, generating 10 kbp long fragments containing the CATCH sequence and separated on agarose gel. Next, the DNA was transferred from the agarose gel to a nitrocellulose membrane by the conventional alkaline method, using the 20× SSC buffer. The radiolabeled probe was generated by a PCR, performed according standard procedure described in Molecular Cloning (Sambrook and Russel 2001), using the radiolabeled α-^32^P dATP (Hartmann Analytic GmbH, Germany) and with primers (catCHStuI and catCHKpnI), specific to the CATCH sequence. Hybridizations were performed at 60 °C in a roller oven (GLF, Germany) and hybridization bottles in the PerfectHyb^Tm^Plus buffer (Sigma-Aldrich, Germany) according to the manufacturer’s protocol. The X-ray film (Kodak BioMax XAR Film, Sigma-Aldrich, Germany) was exposed to the membranes with an intensifying screen at −70 °C for 24 h and developed with the Cerastream Kodak developer solution (Sigma-Aldrich, Germany).

### Northen blot analyses

Equal amounts of total RNA (5 µg), isolated form transformed lineages by the NucleoSpin RNA Plant (Macherey–Nagel, Germany) and separated of 1.2% Formaldehyde Agarose (FA) gel in 1× FA Gel running buffer (100 mL of 10xFA gel buffer (200 mM 3-[N-morpholino]propanesulfonic acid (MOPS), 50 mM Sodium Acetate, 10 mM EDTA, pH = 7) and 20 mL 37% formaldehyde).

The transfer of RNA from the agarose gel to a nitrocellulose membrane was performed by a conventional alkaline method using 20× SSC buffer (3 M NaCl, 0.3 M sodium citrate, pH 7.0). DIG-labeled DNA fragments were prepared by PCR using DIG Probe Synthesis Kit (Roche) with primers catCHStuI and catCHKpnI (hybridizing with the *catCH* sequence) or with primers psbA_F and psbA_R (hybridizing with the *psbA* sequence) and used as hybridization probes. The DIG was detected with alkaline phosphatase (AP)-conjugated anti-DIG antibody (Roche) and CDP-Star (Roche). The X-ray film (Kodak BioMax XAR Film, Sigma-Aldrich, Germany) was exposed to the membranes for 24–48 h and developed with the Cerastream Kodak developer solution (Sigma-Aldrich, Germany).

### Indirect immunofluorescence

*Cyanidioschyzon merolae* cells were grown and synchronized by 12/24 h light/dark regime. Cell fixation and permeabilization was performed, basically as described before (Hoff 2015). *C. merolae* cells were spun down and washed twice with 1x phosphate buffer saline (PBS) pH 7.2 (137 mM NaCl, 2.7 mM KCl, 10 mM Na_2_HPO_4_, 1.8 mM NaH_2_PO_4_). Cells were fixed with 4% formaldehyde for 10 min and washed 3× with 1× PBS buffer. Permeabilization was performed with 0.1% TritonX-100/PBS for 15 min and washed 3× with 1× PBS buffer. Blocking was performed with 5% BSA in 1× PBS for 45 min at RT. Next, cells were incubated at 4 °C overnight with primary antibody in blocking solution, then washed 4× with 1× PBS buffer. Afterwards, cells were incubated for 1 h at RT with secondary antibody in blocking solution and washed with 1× PBS buffer. Cells were next incubated for 1 h with 1 µg/mL Hoechst 33258 dye (Sigma-Aldrich, Germany) diluted in blocking solution and washed 6× with 1× PBS buffer. Cells were resuspended in 1× PBS buffer and placed on polilizyne microscope slides with equal volume of DAKO Fluorescent Mounting Medium (DAKO North America Inc., USA).

Primary and secondary antibodies were used at the following concentrations: 1:100 for rabbit anti-CAT antiserum, 1:100 for Alexa-488 goat anti-rabbit antibody (Thermo Fisher Scientific, USA).

### Fluorescence microscopy

Cells were observed using microscope Nikon A1R MP with set of filters: DAPI (EX 340–380 nm, DM 400 nm BA 435–485 nm) for blue fluorescence of DNA with Hoechst 33258 dye, FITC (EX 465–495 nm, DM 505 nm, BA 515–555 nm) for green fluorescence of Alexa 488 goat anti-rabbit antibody, G-2A (EX 510–560 nm, DM 575 nm, BA 590 nm) for red auto fluorescence of chlorophyll, FITC/TRITC (EX 475–490 nm, DM 500–540 nm, BA 503–530 nm) for green fluorescence of Alexa 488 goat anti-rabbit antibody and red auto fluorescence of chlorophyll at the same time (EX—excitation filter, DM—diachronic mirror, BA—absorption filter).

## Electronic supplementary material

Below is the link to the electronic supplementary material.

Supplementary material 1 (DOC 1499 KB)
