# Supplementary material for: Transformation of the Cyanidioschyzon merolae chloroplast genome: prospects for understanding chloroplast function in extreme environments
Source: Plant Mol Biol. 2016 Oct 28;93(1):171–83. doi: 10.1007/s11103-016-0554-8 (PMC5243890; doi:10.1007/s11103-016-0554-8)
Supplement: Supplementary file 1 — Supplementary material 1 (DOC 1499 KB) [file 11103_2016_554_MOESM1_ESM.doc]

Stable transformation of the *Cyanidioschyzon merolae* chloroplast genome: prospects for understanding chloroplast function in extreme environments.

**Maksymilian Zienkiewicz, Tomasz Krupnik, Anna Drożak, Anna Golke and Elżbieta Romanowska**

From the Faculty of Molecular Plant Physiology, Department of Biology, University of Warsaw, ul. Miecznikowa 1, 02-096 Warsaw, Poland

To whom correspondence should be addressed: Dr. Maksymilian Zienkiewicz, Faculty of Molecular Plant Physiology, Department of Biology, University of Warsaw, ul. Miecznikowa 1, 02-096 Warsaw, Poland. Telephone: 48-225-543-912; FAX: 48-225-543-910; E-mail: maximus@biol.uw.edu.pl

Supplemental Figure S1


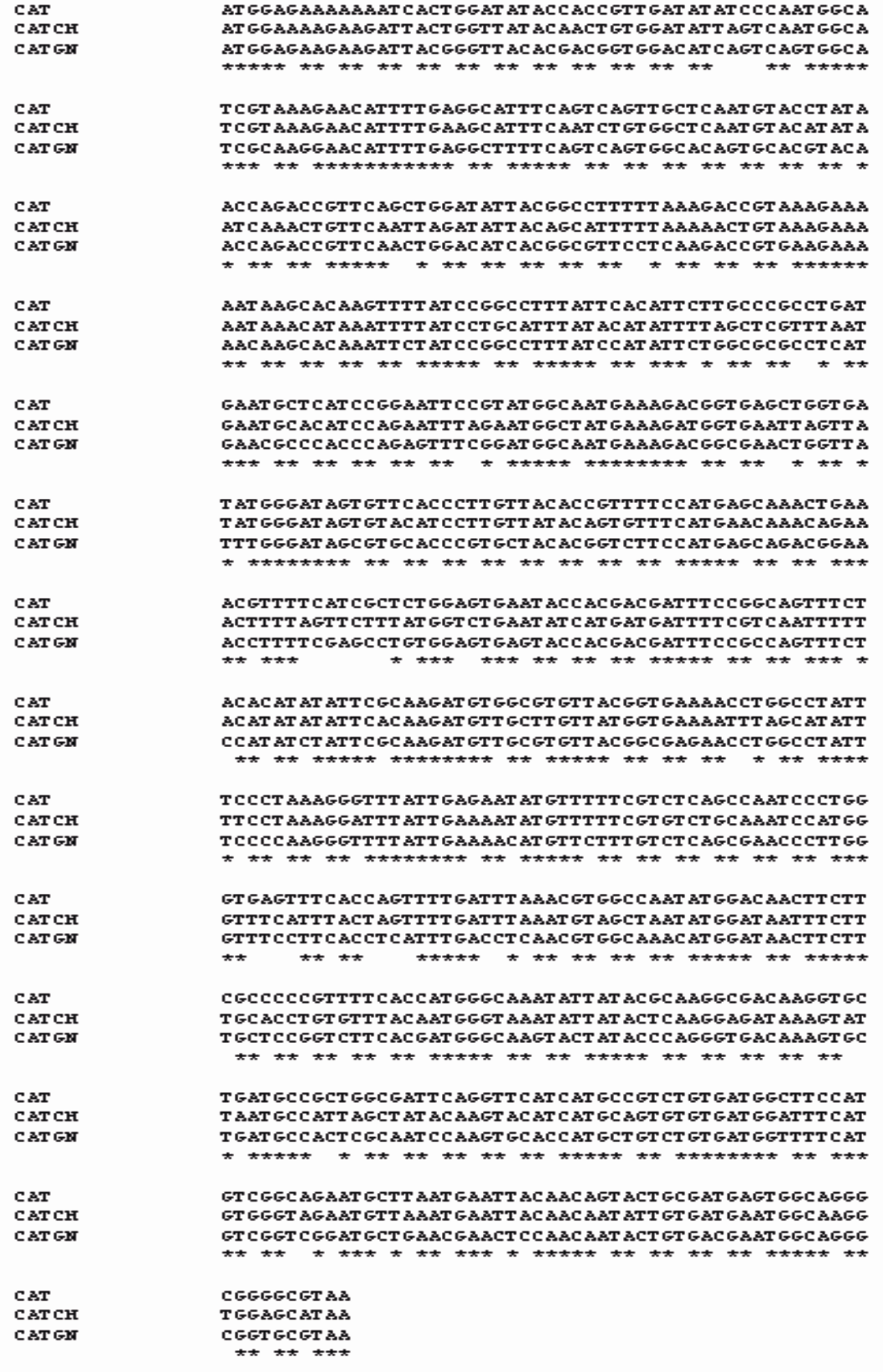


**Fig. S1. The nucleotide sequences of the optimized *cat* gene to chloroplast (CATCH) and cytosolic (CATGN) expression aligned with the native *cat* gene of Tn9 from pACYC184 vector (CAT).** The identity level of bacterial type *CAT* *vs* *CATCH* is 79% (521/660), *CAT vs CATGN* is 80% (531/660), *CATCH vs CATGN* is 75% (498/660). Alignment was generated by T-coffee server (http://www.ebi.ac.uk/Tools/msa/tcoffee/).

Supplemental Figure S2


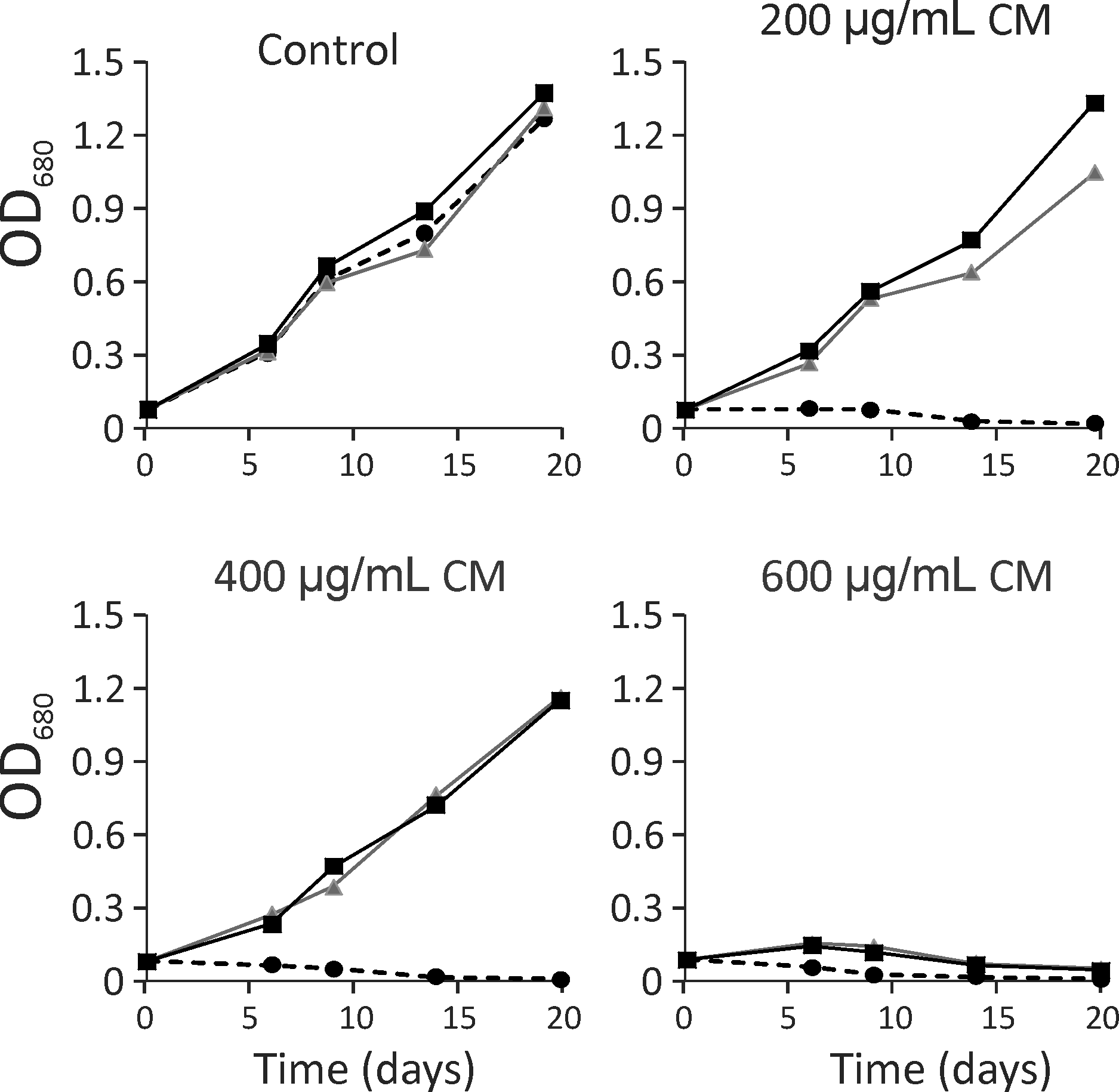


**Fig. S2. Growth dynamic of pCCATCH mutants obtained by biolistic bombardment (B; dark, square marker) and PEG-mediated method (P; gray, triangle marker) *vs*. wild type (WT; black, dashed line) *C. merolae* in rising concentration of chloramphenicol (0-600 μg/mL) after six month of cultivation under chloramphenicol pressure.** Cultures were led for 30 days, under continuous light of 50 μM of photons m^-2^ s^-1^. OD measurements were taken at λ680 nm. Stable chloramphenicol mutants could sustain growth in 400 μg/mL but not in 600 μg/mL of chloramphenicol. Wild type cells growth was inhibited in all tested chloramphenicol concentrations.

Supplemental Figure S3

**
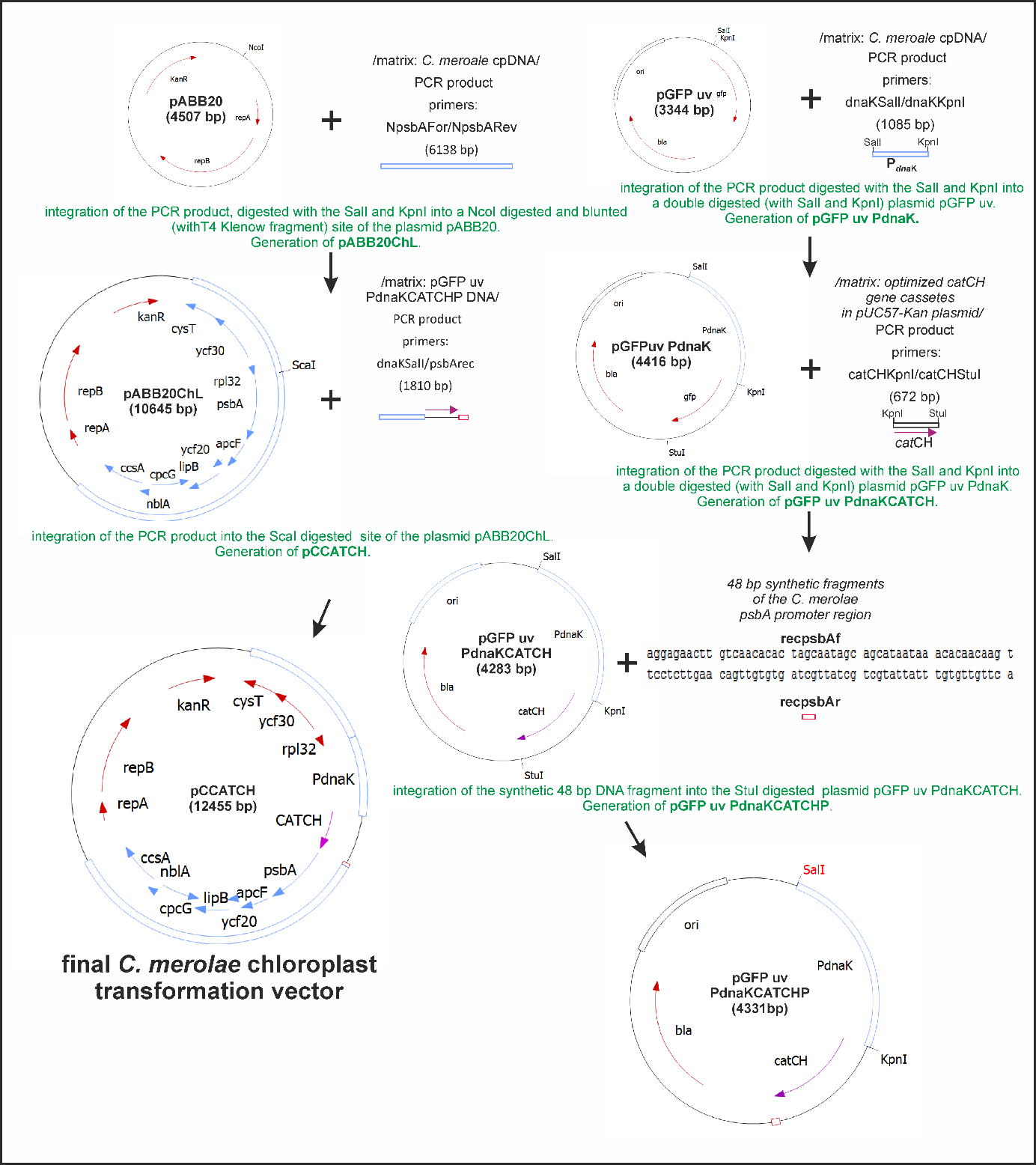
**

**Fig. S3. The construction scheme of the pCCATCH transformation vector.**

Supplemental Figure S4


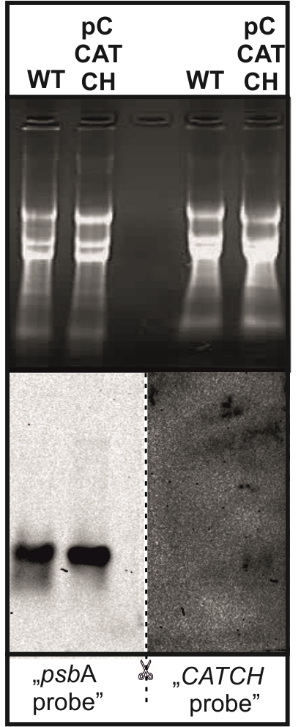


**Fig. S4. Northern blot analysis.** Equal amounts of total RNA (5 µg), isolated form the transformed lineages and the WT were separated on FA-agarose gel and transfer onto a nitrocellulose membrane. The membrane was cut in half and both parts ware probed for *psbA* or *cat* mRNA. It was observed that the position of psbA mRNA (detected in WT and transformant lineages) differed firm the position of *cat* mRNA (detected in the transformant lineages only and marked with an arrow), showing that cat mRNA was transcribed on a different strand than *psbA* and the cat gene possessed functional terminator of transcription.

Supplemental Table S1

**Table S1. List of primers used in this study**. In bold restriction sites used for cloning.

| Name | Sequence 5`→3` |
| --- | --- |
| NpsbAFor | AATGCACCAAATTCGCCCAC |
| NpsbARev | TAAACCCTCCGCTGGTCAAC |
| dnaKSalI | CTCT**GTCGAC**ACAGACAGGATCTGGAAGGG |
| dnaKKpnI | TGGTT**GGTACC**GAACTGTTCTCCTTATGTTGCTTTTAT |
| recpsbAf | AGAACTTGTCAACACACTAGCAATAGCAGCATAATAAACACAACAAGT |
| recpsbAr | CTTGTTGTGTTTATTATGCTGCTATTGCTAGTGTGTTGACAAGTTCT |
| psbArec | ACTTGTTGTGTTTATTATGCTGCTA |
| 5UTF | GTGAGCAACTTCAAGCTAGAACTAAGTAG |
| 5UTR | CGTGTCTGCAAATCCATGGGTTTC |
| 3UTF | TGCTCCACCTTGCCATTCATCAC |
| 3UTR | ATAGGGCAAATTTCTACTAATTATC |
| catCHStuI | **AGGCCTT**TATGCTCCACCTTGCCAT |
| catCHKpnI | **GGTACC**ATGGAAAAGAAGATTACTGGTTATA |
| pABB20L | CCTATTATAGCGGGAGTG |
| pABB20R | GGCTCAGTGACTCTTATC |
| psbA_F | GCTAATTTATGGGAACGTTTTTG |
| psbA_R | ACAGACGGAGCTGTTAATGC |
